# Integrating genome-wide association and transcriptome analysis to provide molecular insights into heterophylly and eco-adaptability in woody plants

**DOI:** 10.1093/hr/uhad212

**Published:** 2023-11-17

**Authors:** Yanmin Hu, Feng Tang, Dan Zhang, Shihua Shen, Xianjun Peng

**Affiliations:** Key Laboratory of Plant Resources, Institute of Botany, The Chinese Academy of Sciences, Beijing 100093, China; Key Laboratory of Plant Resources, Institute of Botany, The Chinese Academy of Sciences, Beijing 100093, China; Key Laboratory of Plant Resources, Institute of Botany, The Chinese Academy of Sciences, Beijing 100093, China; Key Laboratory of Plant Resources, Institute of Botany, The Chinese Academy of Sciences, Beijing 100093, China; Key Laboratory of Plant Resources, Institute of Botany, The Chinese Academy of Sciences, Beijing 100093, China

## Abstract

Heterophylly is regard as an important adaptive mechanism in response to different environments within plants. However, the genetic mechanisms responsible for heterophylly in woody plants are still poorly understood. Herein, the divergence of heterophyllous leaves was investigated at morphogenesis and using microdissection and physiological indexes in paper mulberry, and the genetic basis of heterophylly was further revealed combined with genome-wide association study (GWAS), transcriptome analysis and weighted gene coexpression network analysis (WGCNA). Our results revealed that the flavonoid content and antioxidant activity increased gradually from the entire leaf to the palmatisect leaf, while the hormone content and net photosynthetic rate decreased. Through GWAS and transcriptome analysis, a total of 98 candidate genes and 2338 differentially expressed genes associated with heterophylly were identified. Importantly, we uncovered critical variations in the candidate genes *Bp07g0981* (*WOX*) and *Bp07g0920* (*HHO*), along with significant differences in haplotypes and expression levels among heterophyllous leaves. Our results also suggested that the genes involved in hormone signaling pathways, antioxidant activity, and flavonoid metabolism might be closely related to the heterophylly of paper mulberry, which could account for the physiological data. Indeed, *CR*-*wox* mutant lines showed significant changes in leaf phenotypes, and differential expression profile analysis also highlighted the expression of genes related to phytohormones and transcription factors. Together, the genetic variations and candidate genes detected in this study provide novel insights into the genetic mechanism of heterophylly, and would improve the understanding of eco-adaptability in heterophyllous woody plants.

## Introduction

The leaf is an important organ for photosynthesis, respiration, and sensing the external environment [1, 2]. In the natural world, the shape, size, and color of leaves exhibit a staggering diversity [3, 4]. Actually, the morphology of plant leaves is closely related to strict internal genetic regulation and external environmental changes [2, 5], and the genetic mechanism of leaf morphology has been the focus of leaf development. In recent years, the heterophylly of plants has attracted more and more attention, which refers to the alteration of leaf forms occurring in different environmental conditions, especially light intensity, water availability, ambient temperature, and hypoxia [6, 7]. Heterophylly is an adaptive strategy in various plants, and a preliminary understanding of the formation mechanism of heterophylly has been established through morphological studies, microdissection data, and physiological studies [8].

Heterophylly is widespread among aquatic and amphibious plants. Morphological and anatomical structures were obviously different between submerged leaves and terrestrial leaves [6, 9]. A study in *Rorippa aquatica* has shown that submerged leaves are pinnately dissected whereas terrestrial leaves are simple with serrated margins [9]. *Hygrophila difformis* develops simple serrated leaves in the terrestrial environment, whereas it forms highly complex leaves under the submerged environment [10]. In another heterophyllous plant, *Ranunculus trichophyllus*, the submerged leaves are thin and cylindrical whereas the terrestrial leaves are thick and broad [11]. What is more, it is worth noting that heterophylly also occurs in various land plants, including *Populus euphratica* [12, 13], *Syringa oblata* [14], and *Ginkgo biloba* [15]. The leaf morphology of *P. euphratica* presents a spatial distribution pattern, and the four types of heterophyllous leaves from the bottom to top are linear, lanceolate, ovate, and broadly ovate [16]. The structure and physiology data of *G. biloba* suggest that the photosynthetic capacity and stomatal conductance of the wider long-shoot leaves are both greater than those of short-shoot leaves [15], while shade trees in *S. oblata* have significantly higher specific leaf weight than sun trees [14].

Previous studies have revealed that environmental factors, plant hormones, and transcription factors could regulate the heterophylly of plants [17]. Light, humidity and temperature are the most critical environmental factors regulating heterophylly [11]. For example, ambient temperature is a crucial inducing factor of heterophylly in *R. aquatica*, which develops simpler-formed leaves at 30°C and dissected leaves at 20°C [18]. What is more, phytohormones also play important roles in determining the heterophylly of plants, including auxins (IAAs), gibberellins (GAs), abscisic acid (ABA), and ethylene [17, 19]. A study has revealed that ABA and ethylene can control the heterophylly of *R. trichophyllus*, which indicates that aquatic leaves have higher levels of ethylene and lower levels of ABA [11]. In another study, GA, ABA and ethylene were key regulators of the formation of submerged leaves in *Callitriche palustris* [20]. Transcription factors are also closely connected with leaf development and morphological diversification [21], especially KNOTTED-like homeobox (KNOX), WUSCHEL-related homeobox (WOX), auxin response factor (ARF), homeodomain-leucine zipper (HD-ZIP), KANADI (KAN), YABBY (YAB), and so on. It has been demonstrated that the KNOX–GA module is involved in regulating the heterophylly of *R. aquatica* [9]. Another study has also suggested that ethylene and ABA could regulate heterophylly by modifying the expression of *KANADI* and *HD-ZIP III* [11]. Based on an increasing number of studies in aquatic and amphibious plants, we already have a certain understanding of the molecular basis of heterophylly [21], whereas the regulation mechanisms of heterophylly in woody plants are largely unknown.

Recently, with the rise of sequencing technology, GWAS analysis, transcriptome analysis, and proteome techniques have also been applied to elucidate the genetic mechanism of heterophylly in plants. For example, GWAS analysis has detected 340 significant single-nucleotide polymorphisms (SNPs) and 21 unique genes associated with heterophylly, including the transcription factor MYB, the auxin response factor 19, and cytochrome P450 [22]. Another GWAS analysis in *P. euphratica* identified a series of candidate genes participating in auxin, shape, and stress resistance, and the results suggest that auxin response factor17-like (*ARF17*) is involved in the regulation of heterophylly [23]. Besides, transcriptomic analysis of *C. palustris* identified 4275 differentially expressed genes (DEGs) between aquatic leaves and terrestrial leaves, which suggests that phytohormones and diverse transcription factors are involved in regulating the heterophylly of *C. palustris* [20]. A total of 56, 73, and 222 differential abundance proteins have been determined in different leaf groups through iTRAQ-based proteomic analysis in the heterophyllous woody plant *P. euphratica*, and differential abundance proteins are involved in photosynthesis, primary metabolite accumulation, and stress tolerance [24]. However, existing research has not been enough to uncover the eco-adaptive molecular mechanism of heterophyllous woody plants.

In the present study, leaf type investigation and whole-genome re-sequencing were performed in a natural population of paper mulberry (*Broussonetia papyrifera*), which is an important heterophyllous woody plant from Moraceae with abundant leaf type diversities and high economic values in papermaking, forage, medicine, and so on [25]. To elucidate the genetic architecture of heterophylly in paper mulberry, we further applied deep re-sequencing analysis, genome-wide association analysis, transcriptome analysis and CRISPR/Cas9-mediated mutagenesis. In combination with microdissection data, physiological parameters, and gene expression pattern analysis, the potential regulation mechanism of heterophylly in woody plants is discussed, providing new insights into the study of heterophylly and eco-adaptability in woody plants.

## Results

### Heterophylly in a natural population of paper mulberry

**Figure 1:**
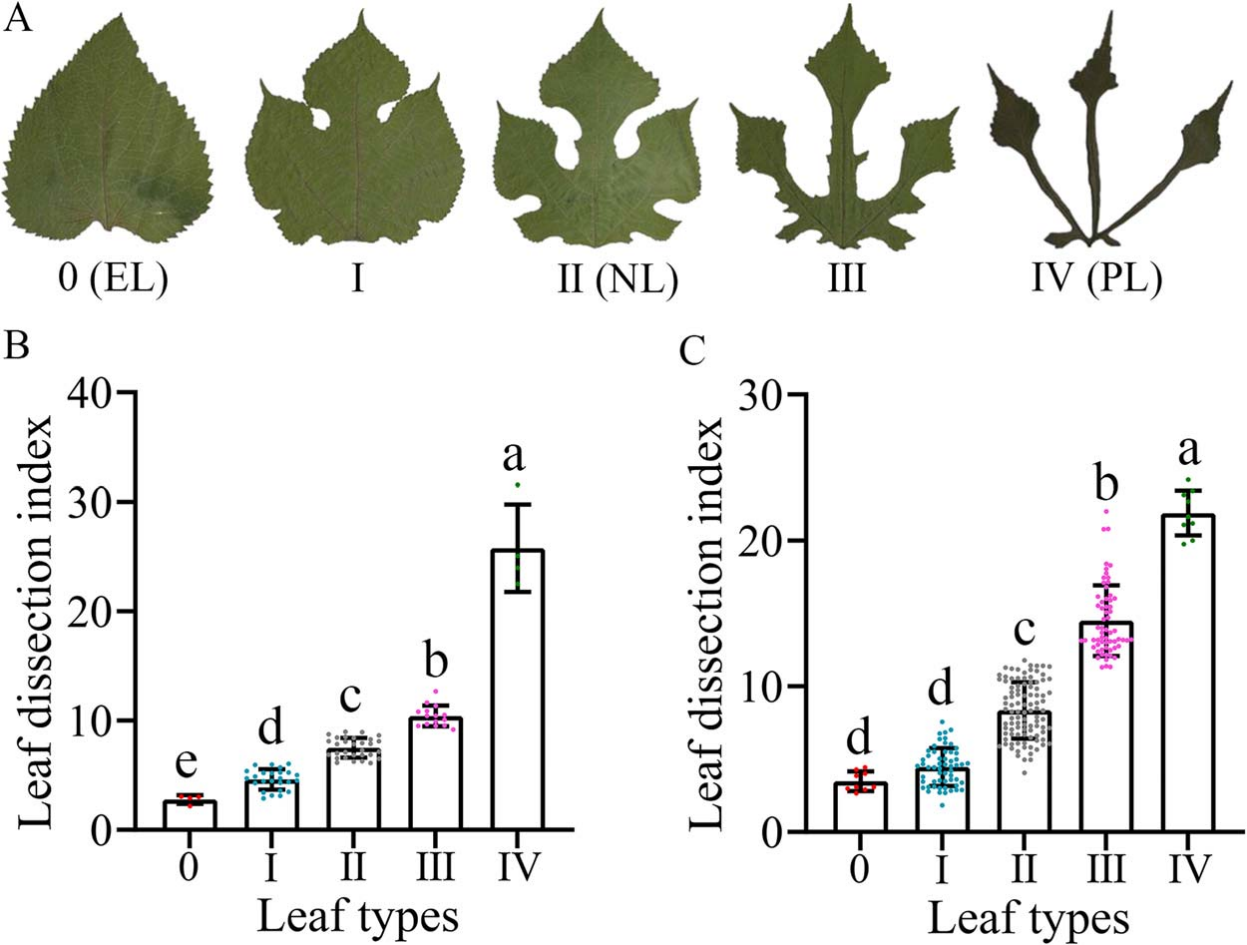
Leaf type survey of paper mulberry. **A** The five leaf types in paper mulberry. EL, entire leaf; NL, natural leaf; PL, palmatisect leaf. **B** Leaf dissection index of each leaf type in paper mulberry from experimental field A (Haidian, Beijing, China). Individual data points are shown together with the means (± standard deviation). Different letters indicate significant differences as determined using ANOVA followed by Tukey’s test (*P* < 0.05). **C** Leaf dissection index of each leaf type in paper mulberry from experimental field B (Shunyi, Beijing, China).

To evaluate the variation of heterophyllous leaves, leaf types (LT) were investigated and analyzed comprehensively and systematically under two different environments, including 78 diverse paper mulberry individuals from 19 sampling sites in experimental field A and 92 diverse paper mulberry individuals from 20 sampling sites in experimental field B (Supplementary Data Table S1). According to a survey of leaf traits from the two experimental fields, we found that the leaves of paper mulberry had split incisions, the number of lobes was variable, and the degree of lobe depth was also different (Fig. 1A, Supplementary Data Figs S1 and S2). Therefore, paper mulberry has typical heterophylly and is an ideal material for studying the genetic mechanism of heterophylly in woody plants.

In this study, the mature leaves of paper mulberry were roughly divided into five types, from entire leaves (type 0) to palmatisect leaves (type IV) (Fig. 1A). By measuring the length, width, perimeter, and area of mature leaves, a quantitative analysis of heterophyllous leaf variations was also conducted based on the leaf dissection index. The results indicated that the leaf dissection indexes of heterophyllous leaves were significantly different; the entire leaf had the minimum leaf dissection index (2.89) while the leaf dissection index of palmatisect leaves (type IV) was maximal (25.88) (Fig. 1C and Supplementary Data Table S2).

### Morphology characteristics and physiological index analysis

**Figure 2:**
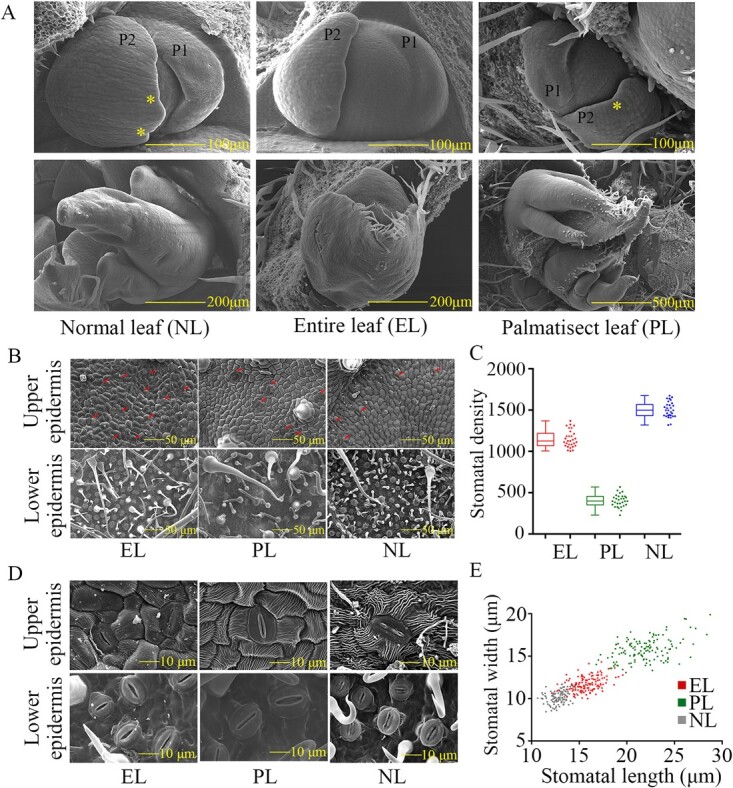
Leaf primordium and stomatal morphology of different types of leaves in paper mulberry. **A** Leaf primordium of the entire leaf (EL), palmatisect leaf (PL), and natural leaf (NL) from three paper mulberry plants. The top pictures show the initial stage of the leaf primordium and the bottom pictures show the developmental stage of the leaf primordium. P1 represents the youngest primordium from the shoot apical meristem. P2 represents the second youngest primordium, and the star represents the small depression. **B** Stomatal density in mature leaf of different leaf types. The arrows point to the locations of the stomas. **C** Statistical results of stomatal density in lower epidermis. **D** Morphology and size of stomas in mature leaves of different leaf types. **E** Scatter diagram of stomatal length and width of different types of leaf.

In order to better understand the heterophylly of paper mulberry, scanning electron microscopy observation was performed for three representative leaf types, comprising the entire leaf (EL), the palmatisect leaf (PL), and the natural leaf (NL) (Fig. 1A). At leaf primordium initiation, the youngest primordium (P1) from the shoot apical meristem was not obviously different among EL, PL, and NL. The edges of the youngest primordium (P1) were both smooth and there was no depression (Fig. 2A). The second youngest primordium (P2) of NL and PL showed a small depression (Fig. 2A), which was the location of nicks of mature leaves, and this result indicated that the leaf types of paper mulberry were already established at the initial stage of leaf primordium. During the development of the leaf primordium, the leaf primordium of both NL and PL was unfurled, with three lobes for each leaf; it could be observed that the morphology of the leaf primordium was basically the same as the shape of mature leaves, while the difference was that the nick of PL was deeper and the lobes were narrower than NL (Fig. 2A). However, there were never nicks in the leaf primordium of EL, and the adjacent leaf primordium presented a state of mutual encapsulation (Fig. 2A).

Because plants depend on stomata for both transpiration and photosynthesis, and plants can adjust their stomatal density and size to adapt to different environments, we also investigated the stomatal morphology and density of EL, PL, and NL (Fig. 1A). The results showed that there was the highest stomatal density (SD) in the lower epidermis of NL, and the stomatal density of EL was 2.8 times higher than that of PL (*P* < .001, Student’s *t*-test) (Fig. 2B and C and Supplementary Data Table S2), whereas stomatal length (SL), stomatal width (SW) and stomatal area (SA) of PL were both significantly higher than EL (*P* < .001, Student’s *t*-test) (Fig. 2D, 2E, Supplementary Data Fig. S3 and Supplementary Data Table S2). The stomatal pore area index (SPI) of EL was higher than that of PL (Supplementary Data Table S2); this is an important index reflecting the potential photosynthetic capacity of plants, and this result indicated that the photosynthetic capacity of EL might be slightly stronger than that of PL. Moreover, the comparison of photosynthetic parameters between EL and PL showed that stomatal conductance, net photosynthetic rate, and transpiration rate in EL were significantly higher than in PL (Supplementary Data Fig. S4 and Supplementary Data Table S2), which reflected that EL had higher photosynthetic capacity, and this result was consistent with the SPI index.

**Figure 3:**
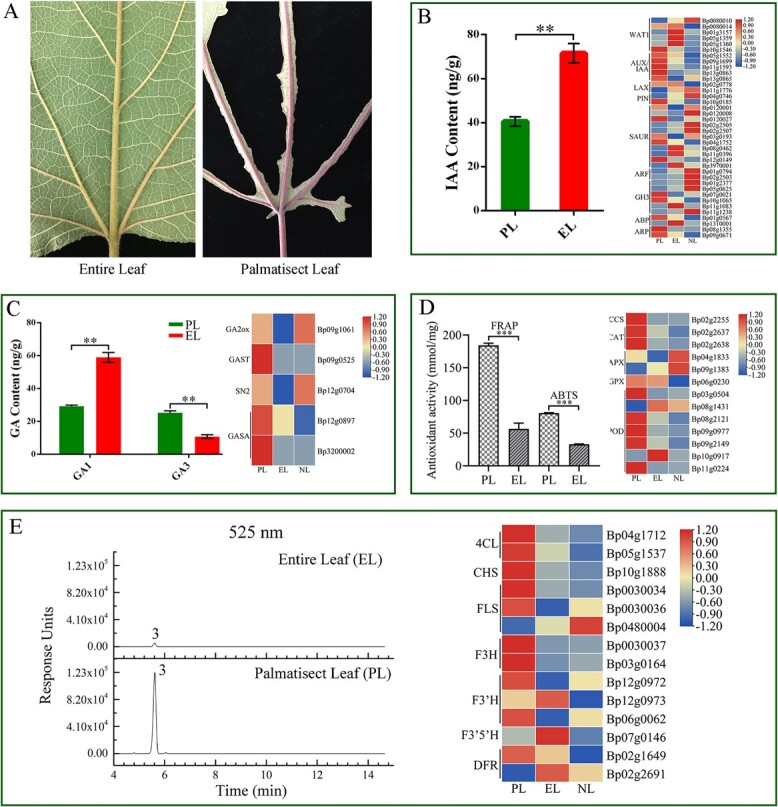
Key physiological indicators and gene expression patterns between entire leaf (EL) and palmatisect leaf (PL). **A** Difference in leaf type and leaf vein color between mature leaves of EL and PL. **B** IAA content in EL and PL (left), and expression patterns of genes involved in the auxin signaling pathway (right). Asterisks indicate significant differences as analyzed by Student’s *t*-test (**P < 0.01). **C** Analysis of the GA content in EL and PL (left) (**P < 0.01), and expression patterns of genes involved in the GA signaling pathway (right). **D** Antioxidant activity of EL and PL (left), and expression patterns of genes involved in antioxidant activity (right) (***P < 0.001). **E** UPLC chromatograms of EL and PL at 525 nm (left), and expression patterns of genes involved in anthocyanin biosynthetic pathways (right).

Secondary metabolites are important for plant adaptation to different environments and survival. Our investigation of heteromorphic leaves found that the petioles and veins of EL were both green, while the petioles and veins of PL were both purple-red (Fig. 3A). To expound the color difference of petioles and veins in heterophyllous leaves, qualitative assessment and quantitative analysis of flavonoids were also performed in this study. The total flavonoid content of PL (14.44 mg/g) was higher than that of EL (6.08 mg/g) and NL (14.26 mg/g). A total of 21 compounds were identified from the three materials (Supplementary Data Table S3), which mainly included smaller phenolic acids (peaks 1 and 2), flavonoid glycosides (peaks 4–7), anthocyanins (peak 3), and flavonoids (peaks 8–21) (Supplementary Data Table S3 and Supplementary Data Fig. S5). Among them, 14 and 19 compounds were isolated from EL and NL, while all 21 compounds were identified from PL. Furthermore, quantitative analysis showed that 21 compounds in the three types of leaves (EL, PL, and NL) were different, especially neochlorogenic acid, anthocyanins, vitexin, and apigenin (Supplementary Data Figs S5 and S6).

### Comparative transcriptome analyses for heterophyllous leaves

To further understand the regulation mechanism of heterophylly, transcriptome sequencing was performed on EL, PL, and NL (Fig. 1A and Supplementary Data Table S4). After pairwise comparison of the three materials, a total of 2,338 differentially expressed genes (DEGs) were identified with the threshold of |log_2_(fold change)| ≥ 1 and adjusted *P*-value ≤ 0.05 (Supplementary Data Figs S7A and B). The expression heat map showed that some genes were specifically expressed in different types of leaf (Supplementary Data Fig. S7C), which suggested that these DEGs might play important roles in the regulation of heterophylly in paper mulberry. Then, KEGG (Kyoto Encyclopedia Genes and Genomes) pathway analysis of the DEGs was performed; the result suggested that some DEGs were enriched in the plant hormone signal transduction, phenylpropanoid biosynthesis, flavonoid biosynthesis, and MAPK (mitogen-activated protein kinase) signaling pathways (Supplementary Data Fig. S7D).

Considering that hormones play crucial roles during leaf development, we measured the content of endogenous hormones [auxin (indole-3-acetic acid, IAA) and GA] in the first unfolded young leaves of PL and EL, and the expression levels of genes involved in hormone signaling pathways were also analyzed. The results indicated that the content of IAA in EL was higher than that in PL, while the expression levels of auxin transport and auxin-response genes were significantly different among PL, EL, and NL (Fig. 3B). Measurement of GA content in PL and EL was also performed using GC–MS, and the result showed that two kinds of active GAs (GA1 and GA3) were present in the leaves of paper mulberry. The total content of GA in PL was 55.78 ng/g, and the contents of GA1 and GA3 were similar, while the total GA content in EL was 78.11 ng/g, and the content of GA1 (65.03 ng/g) was significantly higher than GA3 (13.08 ng/g) (Fig. 3C). Meanwhile, the RNA-seq analysis reflected that the GA biosynthetic and metabolic genes were also differentially expressed in PL and EL, including *GA2ox*, *GAST*, *SN2*, and *GASA* (Fig. 3C).

What’s more, the antioxidant activity of PL was significantly higher than that of EL through two detection methods, and PL had higher expression levels of genes involved in the redox signaling system than EL, especially the genes encoding CCS (copper chaperone for superoxide dismutase), CAT (catalase), GPX (glutathione peroxidase), and POD (peroxidase) (Fig. 7D). Further qualitative assessment and quantitative analysis of flavonoids revealed that the content of anthocyanin in PL was significantly higher than in EL (Fig. 3E, Supplementary Data Fig. S6 and Supplementary Data Table S3). Moreover, the expression levels of key genes in the anthocyanin synthesis pathway were significantly different between the two materials, and the genes encoding 4CL, CHS, FLS, F3H, and F3′H exhibited higher expression levels in PL (Fig. 3E), which might be the important reason for the higher content of anthocyanins and purple-red veins of PL.

### Genome-wide association study of leaf type and leaf dissection index

To identify key genes regulating heterophylly of paper mulberry, a set of 170 paper mulberry individuals from different geographical origins were re-sequenced with an average depth of 8.8× (Supplementary Data Table S1). A total of 2,571,952 high-quality SNPs with minor allele frequency (MAF) >0.05 and missing rate <50% were identified after filtering. Based on these high-quality SNPs, we first performed phylogenetic analysis, principal component analysis (PCA), and population structure analysis of the 170 paper mulberry individuals, which could classify the individuals into three major groups (Supplementary Data Fig. S8). Using the generalized linear model (GLM) and the Fixed and random model Circulating Probability Unification (FarmCPU) model, GWAS analyses of the leaf type and leaf dissection index were performed.

**Figure 4:**
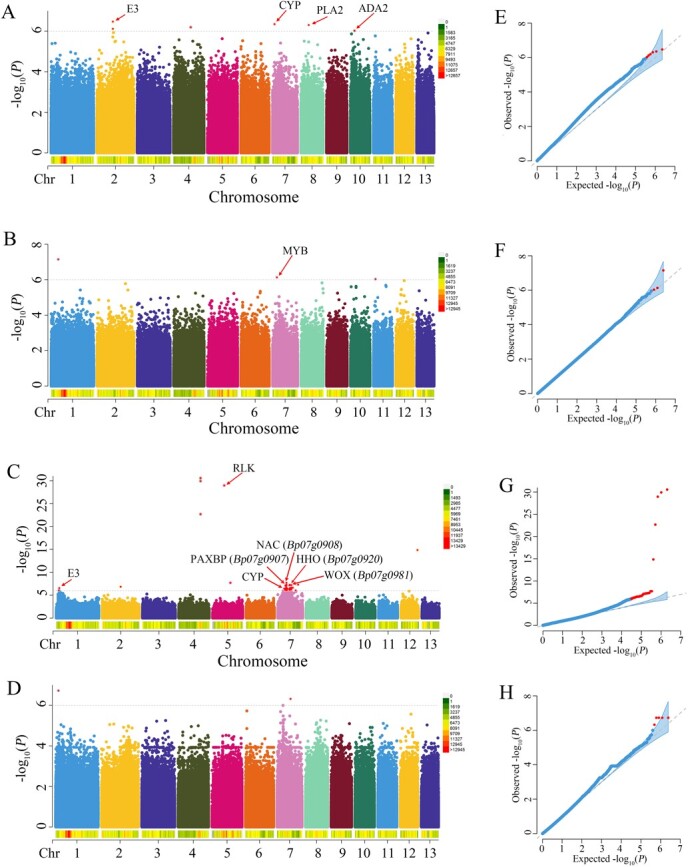
GWAS of leaf type in paper mulberry. **A**, **B** Manhattan plots of GWAS for leaf type from experimental field A using the GLM model and FarmCPU model, respectively. **C**, **D** Manhattan plots of GWAS for leaf type from experimental field B using the GLM model and FarmCPU model, respectively. **D**, **E** QQ plots of GWAS for leaf type from experimental field A using the GLM model and FarmCPU model, respectively. **F** QQ plot of GWAS for leaf type from experimental field B using the GLM model and FarmCPU model, respectively.

In this study, a total of 79 significant SNPs associated with leaf type were detected from two environments (Fig. 4 and Supplementary Data Table S5). Among them, the GLM model and FarmCPU model detected six and three significant SNPs, respectively, through the GWAS of leaf type from experimental field A. For leaf type from experimental field B, there were 65 and 5 significant SNPs identified through the GLM model and FarmCPU model, respectively. Of particular note was 74.7% of the significant SNPs located in chromosome 7. Based on the whole-genome LD decay distance (~10 kb) of paper mulberry from our previous study, 73 candidate genes associated with leaf type were selected for further analysis, including PAX3- and PAX7-binding protein (*Bp07g0907*), NAC transcription factor (*Bp07g0908*), E3 ubiquitin-protein ligase, receptor-like protein kinase (RLK), and cytochrome P450 (CYP) (Supplementary Data Table S5). Of these, the significant SNPs 7:8817651 and 7:9541730 were located in the coding region of *Bp07g0920* and *Bp07g0981*, which encode an MYB-like transcription factor and a WOX transcription factor, respectively.

**Figure 5:**
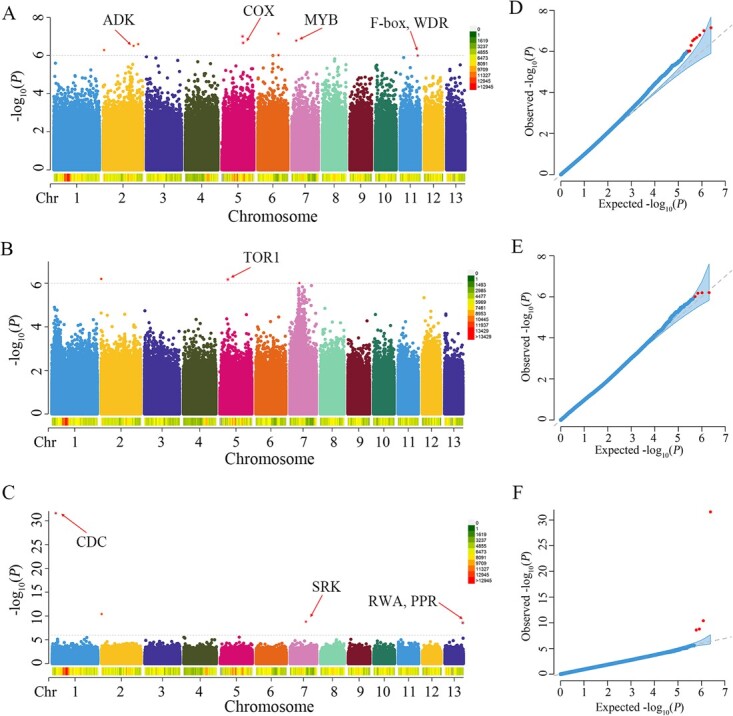
GWAS of the leaf dissection index in paper mulberry. **A** Manhattan plot of GWAS for the leaf dissection index from experimental field A using the FarmCPU model. **B**, **C** Manhattan plots of GWAS for the leaf dissection index from experimental field B using the GLM model and FarmCPU model, respectively. **D** QQ plot of GWAS for the leaf dissection index from experimental field A using the FarmCPU model. **E**, **F** QQ plots of GWAS for the leaf dissection index from experimental field B using the GLM model and FarmCPU model, respectively.

Moreover, 16 significant SNPs associated with the leaf dissection index were also detected (Fig. 5 and Supplementary Data Table S6). Among them, GWAS of the leaf dissection index from experimental field A detected nine significant SNPs using the FarmCPU model, while the GLM model did not detect significant signals. For the leaf dissection index from experimental field B, the GWAS analyses identified three and four significant SNPs using the GLM model and FarmCPU model, respectively. A total of 31 genes were identified as candidate genes for the leaf dissection index, which encoded cytochrome oxidase (COX), WD repeat-containing protein (WDR), cell division cycle protein (CDC), F-box protein and so on (Supplementary Data Table S6). Notably, we also detected two significant SNPs (7:4357706 and 7:9438541) affecting multiple traits, and there were six overlapping candidate genes between leaf type and leaf dissection index, including transcription factor MYB (*Bp07g0490*), MLP-like protein (*Bp07g0973*), and reverse transcriptase (*Bp07g0974*).

**Figure 6:**
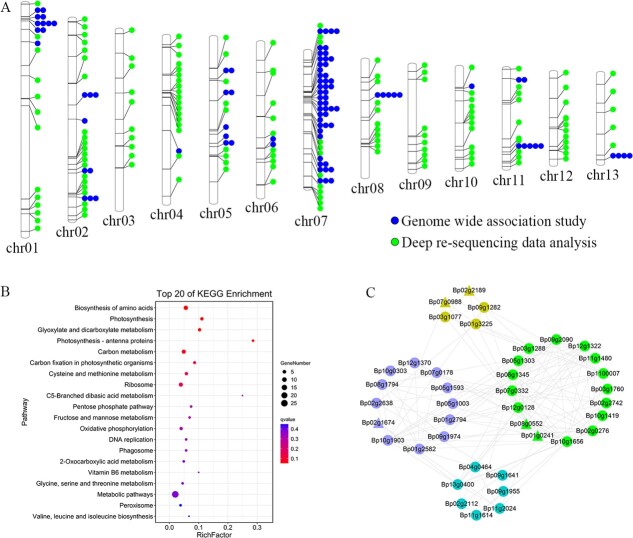
Chromosomal distribution and function analysis of the candidate genes identified in this study. **A** Chromosomal distribution of candidate genes identified by GWAS and variation analysis based on deep re-sequencing data. **B** KEGG enrichment analysis of candidate genes. **C** Protein–protein interaction analysis of candidate genes. The genes identified by GWAS are drawn as triangles and the genes identified by deep re-sequencing data analysis are drawn as circles.

Through the integrated analysis of the genes identified by GWAS and comparative transcriptome analysis, 10 common genes were identified, including *Bp07g0981* encoding a WOX transcription factor, *Bp07g0920* encoding an MYB-like transcription factor (HHO), and *Bp07g0490* encoding transcription factor MYB1R1 (Supplementary Data Fig. S9A and B). The protein–protein interaction analysis result showed that there were close interactions between the proteins encoded by the candidate genes, which were identified by GWAS analysis and comparative transcriptome analysis. The most interesting finding was that Bp07g0981 (WOX transcription factor) could regulate the auxin metabolism pathway through interaction with YABBY (Bp01g1037 and Bp06g0033) (Supplementary Data Fig. S9C), which might be the important regulation mechanism of heterophylly in woody plants. Additionally, deep re-sequencing data analysis was also performed among nine samples with sequencing depth ~20× from EL, PL, and NL, which identified 142 and 48 genes through the SNP and indel variations (missing rate = 0), respectively (Supplementary Data Table S7). The functional annotation results showed that the related genes encoded chlorophyll a and b binding proteins, F-box/kelch-repeat protein, BEL1-like homeodomain protein, transcription factor ERF, Ycf2 and so on. Then, the candidate genes identified by both GWAS and deep re-sequencing data analysis were included in a chromosomal distribution map (Fig. 6A). To better understand the key biological processes involved in these candidate genes, KEGG enrichment analysis was performed, which revealed that photosynthesis, carbon metabolism, biosynthesis of amino acids, and oxidative phosphorylation were the most representative pathways (Fig. 6B). These candidate genes were also used to perform a protein–protein interaction analysis, and the results indicated that the genes in the network map could be divided into four categories: those involved in transcription, translation and posttranslational modification; carbohydrate metabolism and energy conversion; and cytoskeleton and other processes (Fig. 6C).

### Genetic variations underlying the regulation of heterophylly

The subsequent analysis found that the genomic regions of 20 candidate genes contained significant associated SNPs (Supplementary Data Table S8); these were considered the key candidate genes for the heterophylly of paper mulberry, and included *BpHHO* (*Bp07g0920*), *BpWOX* (*Bp07g0981*), *BpPAXBP* (*Bp07g0907*), and *BpNAC* (*Bp07g0908*). Notably, the SNP variants in the coding and promoter regions of the 20 key candidate genes were also explored, and the haplotype analysis of candidate genes revealed important natural variations associated with the heterophylly and eco-adaptability of paper mulberry.

**Figure 7:**
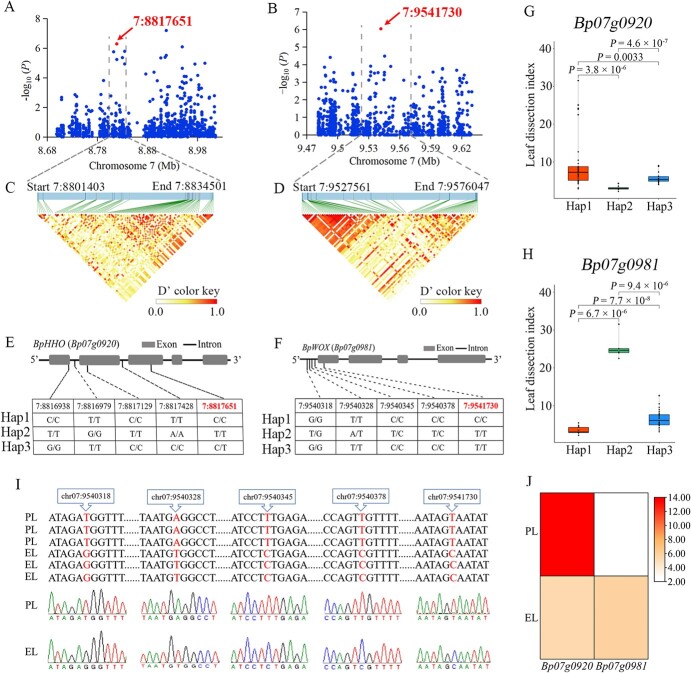
Identification of candidate genes and genetic variations associated with leaf type of paper mulberry. **A**, **B** Local Manhattan plots surrounding the significant SNPs 7:8817651 and 7:9541730. **C**, **D** Linkage disequilibrium heat maps surrounding the significant SNPs 7:8817651 and 7:9541730. **E**, **F** Mutations and haplotype analysis of *Bp07g0920* and *Bp07g0981*. **G**, **H** Box plots with significant differences (*P*-value) showing the leaf dissection index of individuals with different haplotypes of *Bp07g0920* and *Bp07g0981*, constructed by the ggplot package in R (version 4.2.1). **I** PCR and Sanger sequencing of variation sites in the promoter and exon region of *Bp07g0981*. **J** Expression levels of *Bp07g0920* and *Bp07g0981* in PL and EL based on FPKM values.

**Figure 8:**
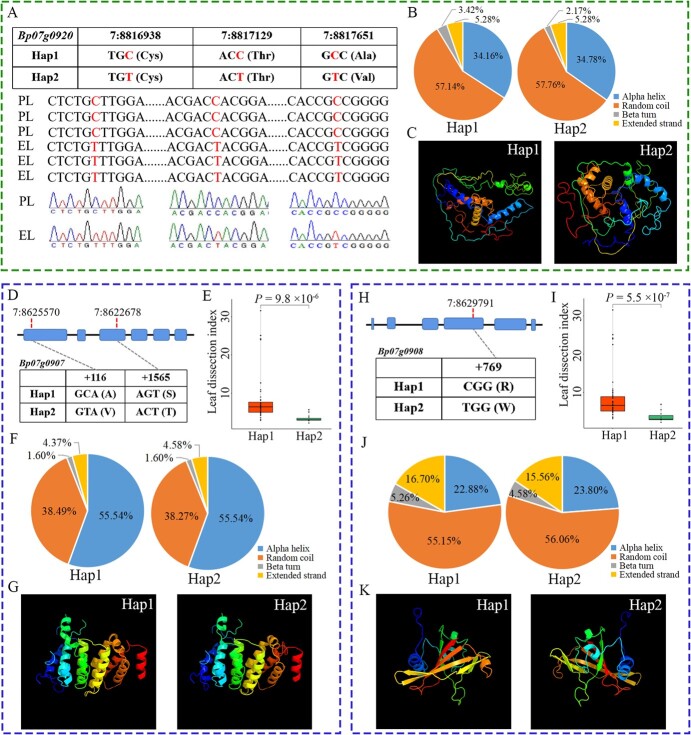
Sequence comparison, mutation sites and haplotype analysis of three candidate genes. **A** Verification of mutations in the exon regions of *Bp07g0920* by Sanger sequencing. **B** Secondary structure prediction results for proteins encoded by different haplotypes of *Bp07g0920*. **C** Tertiary structure prediction results for proteins encoded by different haplotypes of *Bp07g0920*. **D**, **H** Non-synonymous mutations and haplotype analysis of *Bp07g0907* and *Bp07g0908.***E**, **I** Box plots with significant difference (*P*-value) showing the leaf dissection index of individuals with different haplotypes of *Bp07g0907* and *Bp07g0908*, constructed by the ggplot package in R (version 4.2.1). **F**, **J** Secondary structure prediction results of proteins encoded by different haplotypes of *Bp07g0907* and *Bp07g0908*, respectively. **G**, **K** Tertiary structure prediction results of proteins encoded by different haplotypes of *Bp07g0907* and *Bp07g0908*, respectively.

Our re-sequencing data showed that there were five SNPs located in the genomic region of the candidate gene *Bp07g0920* (*BpHHO*, *Hypersensitivity to low phosphate-elicited primary root shortening homolog*), which was a MYB-like transcription factor-encoding gene, and the significant SNP 7:8817651 was located in the third exon of *Bp07g0920* (Fig. 7A and E). We noted significant differences between three major haplotypes and their leaf types (Fig. 7E and G), and the leaf dissection index of the individuals carrying *BpHHO*-Hap1 was significantly higher than that of the individuals carrying *BpHHO*-Hap2. We also verified the SNP sites in *BpHHO* by PCR and Sanger sequencing; the results suggested that the representative leaf materials PL and EL carried different haplotypes; all PLs with the highest leaf dissection index carried *BpHHO*-Hap1 while all ELs carried *BpHHO*-Hap2 (Fig. 8A). Further analysis identified that the significant SNP 7:8817651 was a non-synonymous mutation, and the prediction results showed that the protein secondary structures and tertiary structures of HHO were different between the two haplotypes (Fig. 8B and 8C). Importantly, RNA-seq data showed that the expression level of *BpHHO* (*Bp07g0920*) in PL was higher than that in EL (Fig. 7J).

Besides, the significant SNP 7:9541730 associated with leaf shape, identified on chromosome 7, was located in the first exon of *Bp07g0981*, which is a WOX (WUSCHEL-related homeobox) transcription factor-encoding gene. Further analysis of the re-sequencing data showed that there were also four SNPs located in the promoter region of the candidate gene *Bp07g0981* (~900 bp from the transcription start site) (Fig. 7F). We also found that the individuals with *BpWOX*-Hap2 had significantly higher leaf dissection indexes compared with the individuals carrying *BpWOX*-Hap1 (Fig. 7H). Meanwhile, we further verified the natural variations in *Bp07g0981* by comparing gene sequences between PL and EL using Sanger sequencing. Our results revealed that PL and EL contained different haplotypes, while PL with *BpWOX*-Hap2 had the highest leaf dissection index and EL with *BpWOX*-Hap1 had the lowest leaf dissection index (Figs 1 and 7I). Most importantly, the SNP 7:9540378 was located in an auxin-responsive element (AACGAC) of the promoter region of *Bp07g0981*, and *Bp07g0981* was more highly expressed in EL than PL (Fig. 7J), which reflected that the natural variations in the promoter region might affect the expression level of *Bp07g0981*.

Also, two non-synonymous mutations (7:8625570 and 7:8622678) were located in the first and third exons of the candidate gene *Bp07g0907*, respectively (Fig. 8D), which encoded a PAX3- and PAX7-binding protein. We found that the leaf dissection index of individuals carrying *Bp07g0907*-Hap1 was significantly higher than that of individuals with *Bp07g0907*-Hap2 (Fig. 8E). Most interestingly, the prediction results showed that the non-synonymous mutations changed the secondary structure and tertiary structure of the encoded protein (Fig. 8F and G), which might directly affect the function of *Bp07g0907*. The other candidate gene, *Bp07g0908*, also contained a non-synonymous mutation (7:8629791) in the fourth exon (Fig. 6H), which encoded an NAC domain-containing protein. Further analyses suggested that the predicted protein structures of the two haplotypes were different, and the leaf dissection index between individuals carrying *Bp07g0908*-hap1 and *Bp07g0908*-hap2 was also significantly different (Fig. 8I–K).

### Identification of key modules and genes via weighted gene coexpression network analysis

**Figure 9:**
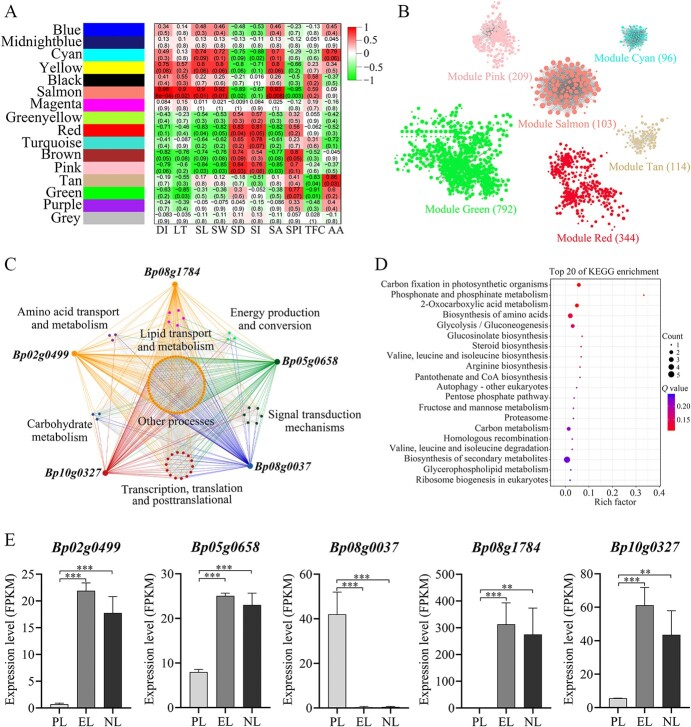
Identification of modules and important genes associated with the physiological phenotype data of paper mulberry. **A** Heat map of correlation coefficient and correlation significance between the modules and different traits. DI, leaf dissection index; LT, leaf type; SL, stomatal length; SW, stomatal width; SD, stomatal density; SI, stomatal index; SA, stomatal area; SPI, stomatal pore area index; TFC, total flavonoid content; AA, antioxidant activity. **B** Gene coexpression network for the top six modules which had higher correlation coefficients and significant correlation. **C** Network map for genes belonging to the salmon module. Associations involving *Bp02g0499*, *Bp05g0658*, *Bp08g0037*, *Bp08g1784*, and *Bp10g0327* are highlighted in different colors. **D** KEGG enrichment analysis among genes in the salmon module. **E** Expression levels of five key genes in the salmon module based on FPKM values. Asterisks indicate significant differences as analyzed by Student’s *t*-test (**P < 0.01, ***P < 0.001). PL, palmatisect leaf; EL, entire leaf; NL, natural leaf; *Bp02g0499*, WD repeat domain phosphoinositide-interacting protein; *Bp05g0658*, G-type lectin S-receptor-like serine/threonine-protein kinase; *Bp08g0037*, UDP-glycosyltransferase; *Bp08g1784*, outer envelope membrane protein; *Bp10g0327*, nuclear transcription factor.

In the present study, a weighted gene coexpression network analysis (WGCNA) was also used to reveal the association between the genes detected from transcriptome analysis and 10 physiological phenotype data. A total of 16 coexpression modules were obtained, and the modules with |correlation coefficient| > 0.8 and *P* < 0.05 were selected as the significant trait-correlated modules. The results suggested that the expression levels of genes within the salmon, red, green, pink, cyan, and tan modules were significantly positively and negatively correlated with the physiological phenotype data (Fig. 9A and B, Supplementary Data Tables S9–S14). Among them, the green and tan modules were significantly negatively correlated with the total flavonoid content (TFC), which indicated that these genes might be closely related to the flavonoid synthesis pathway. The expression levels of genes within the tan module were significantly positively correlated with antioxidant activity. Notably, the salmon module of 103 genes was significantly positively correlated with dissection index, LT, SL, SW, and SA, whereas it was significantly negatively correlated with SD and SPI (Fig. 9A).

Furthermore, the network map for genes belonging to the salmon module suggested that these genes were mainly involved in transcription, translation and posttranslation, signal transduction mechanisms, lipid transport and metabolism, and energy production and conversion (Fig. 9C). Then, KEGG pathway analysis of the genes in the salmon module was performed, and these genes were enriched mainly in biosynthesis of secondary metabolites, biosynthesis of amino acids, carbon fixation in photosynthetic organisms and so on (Fig. 9D). In the salmon module, the top five most connected genes (*Bp02g0499*, *Bp05g0658*, *Bp08g0037*, *Bp08g1784*, and *Bp10g0327*) were considered as prioritized hub genes for further study. Notably, the expression patterns of these genes were also significantly different among heterophyllous leaves (Fig. 9E). RNA-seq data reflected that *Bp02g0499*, *Bp05g0658*, and *Bp10g0327* exhibited higher expression levels in EL and NL. The expression level of *Bp08g0037* was significantly higher in PL than in EL and NL, whereas *Bp08g1784* was only expressed in EL and NL.

Integrated analysis also was performed between the candidate genes from GWAS analysis and the genes from the significant trait-correlated modules of WGCNA (salmon, red, green, pink, cyan, and tan), and eight common genes were detected. Most interestingly, the candidate genes *Bp07g0908* and *Bp07g0920* were contained in the salmon module, which encoded an NAC domain-containing protein and an MYB-like transcription factor, HHO. Both *Bp07g0908* and *Bp07g0920* included important natural variations (Fig. 8), which were the key candidate genes detected by GWAS analysis. What is more, *Bp07g0840* (encoding a membrane protein) was contained in the cyan module, *Bp07g0236* (encoding the protein HHL1), *Bp07g1192* (encoding a transmembrane protein), and *Bp07g1698* (encoding a GDP-fucose protein *O*-fucosyltransferase) were contained in the green module, *Bp02g1673* (encoding a γ carbonic anhydrase) was contained in the red module, and *Bp02g1672* (encoding the protein RER1B) was contained in the tan module.

### Differential gene expression between *CR-wox* and wild-type lines

**Figure 10:**
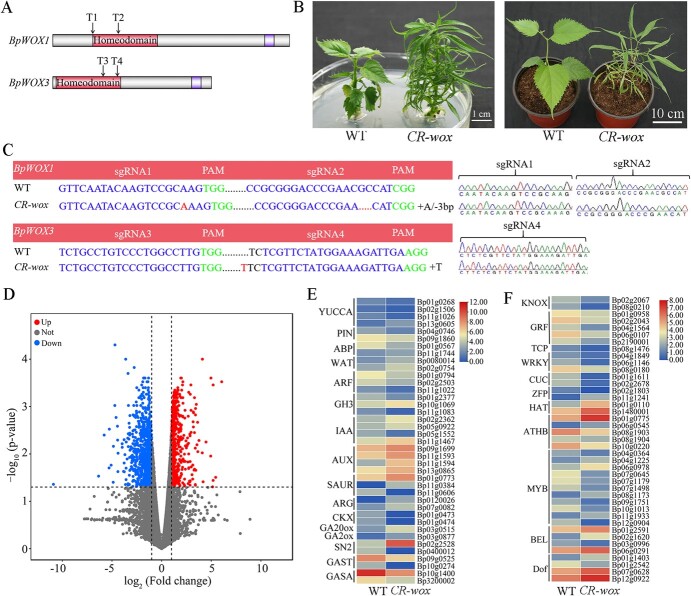
Mutation sites, leaf phenotypes, and DEGs of the paper mulberry WT and CRISPR/Cas9-edited *wox* mutant (*CR-wox*) lines. **A** Schematic diagram of the designed target sites in *BpWOX1* and *BpWOX3* genomic sequences. **B** Phenotypes of the WT and *CR-wox* mutant lines. **C** PCR and Sanger sequencing of editing sites in the WT and *CR-wox* mutant lines. **D** Volcano plot showing significant DEGs between the WT and *CR-wox* mutant lines. **E** Heat map showing expression patterns of DEGs involved in the hormone signaling pathway. **F** Heat map showing expression patterns of DEGs related to transcription factors.

In our study, the function of *WOX* genes from the WUS clade in leaf development of paper mulberry was also verified through the CRISPR/CAS9-mediated mutagenesis of *BpWOX*s. In our previous research on the *WOX* gene family in paper mulberry, we found that *BpWOX1* (*Bp12g1344*), *BpWOX2* (*Bp07g0981*), and *BpWOX3* (*Bp09g1360*) were clustered into the same WUS clade with the similar protein domain [26]. So we designed four sgRNAs targeting the homologous regions of these three WUS-clade *BpWOX* genes and constructed a series of CRISPR/Cas9 vectors for knockout experiments. After the identification of positive lines and phenotypic observation, we detected a double mutant line (named *CR-wox*) with significant phenotypic variation, which appeared as a narrow-leaflet phenotype compared with the wild type (WT) (Fig. 10B). Besides, we observed three insertion/deletion mutations in three target sites between the WT and *CR-wox* mutant line using PCR amplification and Sanger sequencing: a single-nucleotide insertion in the T1 target site (sgRNA1) and a 3-bp deletion in the T2 target site (sgRNA2) within *BpWOX1*, and a single-nucleotide insertion in the T4 target site (sgRNA4) within *BpWOX3* (Fig. 10A and C), which directly verified that *BpWOX*s played important roles in the leaf development of paper mulberry. The qRT–PCR result showed that the expression levels of *BpWOX1* and *BpWOX3* were obviously reduced in the *CR-wox* mutant line (Supplementary Data Fig. S10).

To gain better insight about the molecular mechanism of *BpWOX*s regulating the leaf development of paper mulberry, transcriptome sequencing of *CR-wox* and WT lines was also performed. Differential gene expression analysis between *CR-wox* and WT identified 1,696 DEGs (≥2-fold changes in expression, adjusted *P* value ≤ 0.05) (Supplementary Data Table S15). Among them, 812 genes showed upregulated expression, while 884 genes showed downregulated expression (Fig. 10D). Furthermore, the KEGG pathway analysis suggested that metabolic pathways and biosynthesis of secondary metabolites were the most significantly enriched pathways (Supplementary Data Fig. S11). Also, we found that there were some genes enriched in the plant hormone signal transduction pathway, especially genes related to the auxin and GA signaling pathways.

Our results reflected that the expression of the auxin biosynthetic *YUCCA* genes, the auxin transport component *PIN* genes and the auxin response factor *ARF* genes were reduced, while the expression of auxin-responsive *IAA* genes and auxin-induced *AUX* genes were increased in the *CR-wox* plant (Fig. 10E). Moreover, two cytokinin oxidase genes (*Bp01g0473* and *Bp01g0474*) were highly downregulated, and the expression of GA biosynthesis and response-related genes also were significantly changed in the *CR-wox* plant compared with WT, such as the gibberellin 20 oxidase gene *GA20ox*, gibberellin 2-β-dioxygenase *GA2ox*, and GA-regulated genes *GAST*, *GASA*, and *SN2* (Fig. 10E). What is more, the expression levels of a series of transcription factor genes were also significantly different between the *CR-wox* line and WT, including *KNOX*, *TCP*, *CUC*, *GRF, MYB*, *BEL*, and *Dof* (Fig. 10F). Based on the above results, we propose that *BpWOXs* regulate the leaf development process of paper mulberry mainly through the hormone signaling pathway and transcriptional regulation.

## Discussion

To adapt to the changeable environment, plants have formed a variety of adaptive strategies; altering leaf morphology is one of the important adaptation mechanisms for heterophyllous plants [27, 28]. Although heterophylly has been the subject of a preliminary study in aquatic and amphibian plants [6, 10], the genetic basis for eco-adaptability of heterophyllous leaves is still unclear in terrestrial woody plants, and the relevant studies were mainly carried out in *Populus euphratica* [22, 24, 29]. To further clarify the mechanisms underlying heterophylly in woody plant species, we systematically and comprehensively evaluated the variation of heterophyllous leaves in a natural population of paper mulberry, which exhibits significant heterophylly and eco-adaptability [30]. The leaf types of paper mulberry could be characterized into five major groups, and leaf morphologies were obviously different in the same plant, while the number of lobes and the degree of lobe depth among different plants were also significantly different. Therefore, paper mulberry was an ideal model plant for studying heterophylly and leaf development, just like the heterophyllous woody plant *P. euphratica*.

Previous research has revealed that the photosynthetic capacity of heterophyllous leaves exhibits differences [16, 24]. In our investigation, the differences in net photosynthetic rates, stomatal conductance, and transpiration rate between EL and PL also demonstrated the different photosynthetic capacities of heterophyllous leaves. It is worth mentioning that the different photosynthetic capacities of heterophyllous leaves should be closely related to stomatal density, which could be proved by the significantly higher stomatal density of EL. Referring to the previous studies [16], we propose that the heterophylly of paper mulberry is a potential adaptive mechanism for the complex and unstable environment, such as light intensity. In addition, our results also suggested that the total flavonoid content and compounds were also significantly different among heterophyllous leaves, especially neochlorogenic acid and anthocyanins, which could reflect the different application values of heterophyllous leaves.

The most meaningful finding of this study was the identification of important candidate genes and genetic variations associated with heterophylly by whole-genome re-sequencing and GWAS analysis, especially *Bp07g0981* (*BpWOX*) and *Bp07g0920* (*BpHHO*). WOX family transcription factors have been reported to be key regulators of leaf development, and the function of WOXs in leaf lamina expansion has been verified in various plant species [31–34]. A total of 10 *WOX* genes were identified from paper mulberry; our previous research showed that five *BpWOXs* had transcriptional activity and seven members were uniquely localized to the nucleus, and could be induced by multiple hormones and abiotic stresses [26]. In this study, various data showed that WOX transcription factors play an important role in the regulation of leaf development and heterophylly in woody plants. Four natural variations were identified in the promoter region of *Bp07g0981* (WOX transcriptional regulator), which might directly affect the expression level of *Bp07g0981* and thus regulate the heterophylly of paper mulberry. Besides, the protein–protein interaction analysis suggested that Bp07g0981 could regulate the auxin signaling pathway through interaction with YABBY. More importantly, our transgenic study also demonstrated that *BpWOX*s were crucial regulators for leaf development in paper mulberry. Furthermore, *Bp07g0920* (*BpHHO*) was the common gene identified through GWAS analysis and WGCNA, which encodes an MYB-like transcription factor and plays multiple roles in plant growth and development [35]. A study in *Arabidopsis* showed that *HHO5* (*At4g37180*) can regulate the expression of *WUSCHEL* (*WUS*) transcription factor through interacting with ultrapetala1 (ULT1) [36]. It also was reported that the MYB domain transcription factor AS1 plays a key role in the regulation of leaf initiation and separation from the SAM by stably suppressing the expression of *KNOX* genes [2, 21]. Here, we found that two major haplotypes of *BpHHO* (*Bp07g0920*) were distributed in EL and PL, and the expression level of *BpHHO* (*Bp07g0920*) was also significantly different between the two materials, so we speculated that *BpHHO* might be a possible novel regulator of leaf development and heterophylly in woody plants.

A noteworthy aspect is that multiple phytohormones play critical roles in the regulation of heterophylly. Consistent with this view, the present study showed that there were differences in the content of GA and auxin (IAA) among the heterophyllous leaves of paper mulberry, along with the different expression levels of genes involved in hormone signaling pathways. Auxin is an important regulator of leaf primordium initiation and development [21, 37], while GA can regulate the formation of leaflets and reduce the leaf complexity of plants [9, 38]. So the higher content of auxin and GA in the EL indicated that auxin and GA might promote the horizontal growth of leaves in paper mulberry. On the other hand, our RNA-seq analysis of heterophyllous leaves and the *CR-wox* mutant line both highlighted the expression of auxin-related genes and GA-related genes, including *YUCCA*, *PIN*, *ARF*, *GA20ox*, and *GA2ox*. Our results are consistent with the previous report that *WOXs* can regulate leaf shape and form through controlling the expression of the auxin biosynthetic gene *YUCCA* [39]. Additionally, two cytokinin oxidase genes (*Bp01g0473* and *Bp01g0474*) were highly downregulated in the *CR-wox* mutant line. Cytokinins play diverse roles in regulating leaf development [40, 41], and the relevant research has shown that WOX transcription factors could modulate cytokinin homeostasis during leaf blade development [42], which suggested that *BpWOX*s might regulate leaf development by influencing the cytokinin pathway of paper mulberry.

It should be noted that our study also identified some other key genes through GWAS and WGCNA analyses, such as cytochrome P450 (*Bp07g0752*, *Bp07g0237*), WD repeat-containing protein (*Bp02g0499*), UDP-glycosyltransferase (*Bp08g0037*), and serine/threonine-protein kinase (*Bp05g0658*), which might also be involved in the regulation of heterophylly in woody plants. Consistent with this view, the previous GWAS analysis in *P. euphratica* also identified significant genes associated with heterophylly, which encoded WD repeat-containing protein, serine/threonine-protein kinase [23], and cytochrome P450 [22]. Moreover, this study identified a series of DEGs encoding KNOX, TCP, CUC, BEL, and Dof transcription factors, which are also important for leaf development and heterophylly of plants. The *KNOX1* gene can regulate the heterophylly in *R. aquatica* by influencing the level of GA [9, 43]. A recent report in the heterophyllous plant *Hygrophila difformis* has shown that *HdCUC3* is involved in regulating heterophylly by interacting with *HdSTM* [10], and *CUC*s are necessary for leaflet formation and leaf margin development [44, 45], while the expression of *CUC* genes is negatively regulated by TCP transcription factors [46]. Besides, it is thought that Dof transcription factors can influence the axial patterning of leaves and BEL-like homeodomain proteins can regulate the expression of *KNOX*s in leaf margins [47, 48].

Taken together, the results of our study suggest that WOX transcription factors play an important role in the regulation of leaf development and heterophylly in woody plants, and could interact with other transcription factors and regulate the expression of genes in hormone signaling pathways. Besides, the candidate gene *Bp07g0920* (encoding the MYB-like transcription factor HHO) might be a possible novel regulator of leaf development and heterophylly in woody plants. Other candidate genes will also provide important references for future research on heterophylly, which could improve our understanding of heterophylly and eco-adaptability in woody plants. However, there are few reports of transgenic studies and the functional identification of genes regulating heterophylly, especially in heterophyllous woody plants, which may be due to their long life cycle and the lack of efficient genetic transformation systems. For future study, more transgenic studies should be performed on the candidate genes identified in the present study, and further verify the underlying molecular mechanisms of these genes regulating heterophylly in paper mulberry.

## Materials and methods

### Phenotypic identification of leaf characteristic index

The paper mulberry resources were widely collected from different geographic regions. To obtain reproducible and reliable phenotypic data, we performed seed germination and seedling hardening, then the seedlings were planted in two experimental fields; experimental field A was in Haidian, Beijing, China (40°6′ N, 116°8′ E) and experimental field B was in Shunyi, Beijing, China (40°12′ N, 116°45′ E). Ten seedlings developed from seeds from each sampling site were planted, the row spacing and plant spacing in experimental field A was 1.5 m × 1 m, and the row spacing and plant spacing in experimental field B was 3 m × 2 m. For all plants the same field management of water, fertilizer, and weeds was used, following the basic method.

During the vigorous growth period in August, three mature leaves from each plant were used to make specimens. The leaf specimens were photographed against a white background with a scale of 10 cm, and the leaf correlation indexes were measured using Digimiter software, including leaf length (LL), leaf width (LW), leaf perimeter (LP), and leaf area (LA). The average values of LP and LA were used to calculate the leaf dissection index [DI, DI = LP^2^/(4π × LA)] [49]. According to the leaf characteristic index, the leaves of paper mulberry were classified into five types. The leaf types and average leaf dissection index of each individual were used for subsequent association analyses.

### Scanning electron microscopy and physiological measurement

In this study, the leaf primordium and stomatal morphology of mature leaves were observed by scanning electron microscopy. Fresh terminal buds and mature leaves from three type trees with entire, palmatisect and natural leaves were fixed for >24 h in FAA fixation solution (formaldehyde: acetic acid:50% ethanol = 1:1:18). The fixed material was dehydrated using a graded ethanol series (50, 70, 85 and 95%), 15–20 min per step. Then, isopentyl acetate was used to replace the ethanol, and the materials were examined under a scanning electron microscope (Hitachi S-4800) after drying. For stomatal morphology, stomatal length (SL), stomatal width (SW), stomatal area (SA, SA = 1/4 × π × SL × SW), stomatal density (SD), stomatal index (SI, SI = SW/SL), and stomatal pore area index (SPI, SPI = SL^2^ × SD × 10^−4^) were investigated [50].

Heterophyllous leaves of the two extreme types (EL and PL) were randomly selected for the determination of photosynthetic parameters, plant hormones, and antioxidant activity. The net photosynthetic rate (Pn), transpiration rate (Tr), and stomatal conductance (Cs) were measured using the Li-6400 Portable Photosynthesis System, and the average values from three different trees were used as the final data. Antioxidant activity was measured using two kinds of total antioxidant capacity assay kit with the FRAP (ferric reducing ability of plasma) method and ABTS [2,2′-azino-bis(3-ethylbenzthiazoline-6-sulfonic acid) diammonium salt] method (Beyotime, Beijing, China) according to the producer’s instructions. The first unfolded young leaves at the tip of the stem were harvested for measuring the endogenous IAA (indole-3-acetic acid) and GA by gas chromatography–mass spectrometry (GC–MS) according to reported methods for determination of endogenous acid hormones in plants [51]. The measurement was conducted with a GC-QqQ MS system (7890a-5975b, Agilent, USA), and DB-5MS (30 m × 0.25 mm × 0.10 μm, Agilent, USA) was used as the chromatographic column. The average value of three biological replicates was used as the final data, and statistical differences were compared using Student’s *t*-test with SPSS software (version 22).

The total flavonoid content (TFC) of the three types of mature heterophyllous leaves (EL, PL, and NL) was determined at a wavelength of 510 nm according to the aluminum chloride colorimetric method [52, 53]. Qualitative assessment and quantitative analysis of flavonoids were performed using the UPLC–DAD–ESI–MS/MS (high-performance liquid chromatography equipped with photodiode array detection tandem electrospray ionization mass spectrometry) method according to a previous report [54].

### Whole-genome re-sequencing

The natural population used for re-sequencing was composed of 170 paper mulberry individuals, which were collected from the experimental fields and have been investigated for leaf characteristic indexes. In this study, the cetyltrimethylammonium bromide (CTAB) method was used to extract total genomic DNA from fresh young leaves, and a NanoDrop spectrophotometer (Thermo Fisher Scientific, Waltham, MA, USA) and 1% agarose gels were used to check the quality and quantity of DNA. Then, paired-end sequencing libraries were constructed as in our previous studies [25, 55], and the libraries were sequenced using the Illumina HiSeq X Ten platform.

The clean reads were obtained by removing reads with >10% unidentified nucleotides (N), reads with >10 nucleotides aligned to the adapter, and reads with >50% bases having a Phred quality score <5. Then, the re-sequencing data were mapped to the paper mulberry reference genome [56] using BWA mem (version 0.7.13-r1126) with the command ‘mem -t 4 -k 32 –M’ [57]. SNPs were called by the HaplotypeCaller module in the Genome Analysis Toolkit (GATK, version 3.1.0), and the SNPs were further filtered using the parameters ‘QUAL < 30, QD < 2.0, MQ < 40.0, FS > 60.0, clusterSize = 2, clusterWindowSize = 5’, and SNPs with DP <4 were removed [58]. Finally, SNPs with a minor allele frequency (MAF) >0.05 and missing rate <50% were used for a subsequent association study by Vcftools (version 0.1.14) [59].

To better understand the genetic variations among different heterophyllous leaves, nine samples with sequencing depth ~20× were selected from the EL, PL, and NL for a deep re-sequencing data analysis as follows. First, we removed the adapter sequences and low-quality reads [unidentified nucleotides (N) > 10%, reads with >10 nucleotides aligned to the adapter, and reads with >50% bases having a Phred quality score <5]. Second, the clean data were mapped to the paper mulberry reference genome using BWA mem (version 0.7.13-r1126). Third, the SNPs and indels were called by the HaplotypeCaller module in Genome Analysis Toolkit (GATK, version 3.1.0), the strict standard was used to filter SNPs and indels, and only the SNPs and indels with no missing sites (missing rate = 0) were retained. Fourth, annotation of SNPs and indels was performed according to the paper mulberry reference genome using the package ANNOVAR (version 2013-05-20) [60]. Finally, genes with SNPs and indels in the coding region, 5′-UTR, and 3′-UTR were identified as the candidate genes.

### Population genetic and genome-wide association study

A neighbor-joining (NJ) phylogenetic tree was constructed using the ‘neighbor’ parameter in PHYLIP (version 3.6) [61] with 2,571,952 high-quality SNPs. PCA was carried out using Genome-wide Complex Trait Analysis (GCTA, version 1.92.0) [62]. The ADMIXTURE (version 1.3.0) [63] program was used to perform population structure analysis.

In this study, two models were used to carry out the association analysis for the two traits from the two environments. The first was the generalized linear model (GLM) from the software TASSEL (version 3) [64], and the GLM model was run using the commands ‘perl run_pipeline.pl -fork1 -vcf my.vcf -fork2 -r trait.txt -fork3 -q Q.txt -combine4 -input1 -input2 -input3 -intersect -glm -export glm_output -runfork1 -runfork2 -runfork3’. The second was the Fixed and random model Circulating Probability Unification (FarmCPU) model implemented by GAPIT (version 3), which separately estimates a fixed effect model and a random effect model [65]. Then, the CMplot package in R (version 4.2.1) was used to construct the Manhattan and QQ plots for displaying the results of GWAS analysis. To determine the *P*-value threshold of our GWAS results, the number of effective SNPs was estimated using the GEC program (version 0.2) [66], and the suggestive genome-wide significant cutoff was evaluated as approximately −log_10_(*P*) = 6 (*P* = 1/effective SNP number).

### Investigation of candidate genes and genetic variant analysis

Because the LD decay of the whole genome was confirmed in a previous study as 10 kb [25], the candidate genes were screened in the 10 kb upstream and downstream of the significant SNPs. The chromosomal distribution of the target genes identified through GWAS and deep re-sequencing data analysis was visualized using the online tool PhenoGram Plot (http://visualization.ritchielab.org/phenograms/plot/). The online database STRING (https://cn.string-db.org/) was used to perform protein–protein interaction analysis of the target genes, and the interaction network was displayed using Cytoscape software (version 3.9.1) [67]. KEGG pathway analysis was performed through the online platform (http://www.omicshare.com/tools).

Then, the location and type of SNP mutations were analyzed based on the paper mulberry reference genome, and the non-synonymous SNPs that altered protein coding and the impacted amino acids were selected. To evaluate the effect of non-synonymous SNPs on protein structure, the predictions of protein secondary structure and tertiary structure were performed using the online tools SOPMA and Phyre2 [68, 69], respectively. Then, the paper mulberry individuals were classified into distinct haplotypes according to the re-sequencing data. The phenotypic differences of distinct haplotypes were calculated by Student’s *t*-test, and the ggplot package in R (version 4.2.1) was used to construct box plots with the *P*-value. The LDBlockShow program (version 1.35) was used to analyze the LD blocks around the important significant SNPs [70].

To confirm the genetic variants located in the key genes, three PL samples and three EL samples from six independent plants were selected for genomic DNA extraction, and PCR amplification was performed using specific primers (Supplementary Data Table S16). Then, the amplification products were cloned into the TA/blunt cloning vector and four clones of each sample were selected for Sanger sequencing.

### RNA-seq analysis of heterophyllous leaves

To perform comparative transcriptome analyses for heterophyllous leaves, three representative leaf materials were selected to perform RNA-seq, comprising EL, PL, and NL. The first unfolded young leaves at the tip of the stem were harvested, immediately frozen in liquid nitrogen and stored at −80°C. Each sample collected from three individual perennial plants and two biological replicates were analyzed. Total RNA was extracted using the TransZol reagent (TransGen, Beijing, China). Then, the construction of a RNA-seq library was completed at Annoroad Gene Technology (Beijing, China) and the library was sequenced on an Illumina NovaSeq PE150 platform. The clean reads were mapped to the paper mulberry reference genome through TopHat software (version 2.0) [71]. Gene expression levels were counted and normalized into fragments per kilobase per million mapped reads (FPKM) values for all samples.

The differential expression analysis among the three leaf materials (EL versus NL, PL versus NL, EL versus PL) was perform using the EBSeq package in R (version 4.2.1) [72]. DEGs were screened with the standard of |log_2_(fold change)| ≥ 1 and the adjusted *P*-value ≤0.05. KEGG pathway analysis and heat map drawing were also conducted with the DEGs.

### Weighted coexpression network analysis

In this study, the gene coexpression network was constructed based on the RNA-seq date of EL, PL, and NL by using the WGCNA (weighted gene coexpression network analysis) package in R software (version 4.2.1) [73]. The phenotype and physiological data were also imported into the WGCNA package, while the Pearson’s correlation coefficients were calculated and the color depth of the heat map was used to evaluate the correlation between the gene modules and phenotype data. Then, the Cytoscape formats with edge weights of each module were exported from the WGCNA package. The coexpression edges with weights >0.1 were retained, and the gene network was visualized through the Cytoscape program (version 3.9.1). The connections of each gene were indicated by their degree in the network, and the top five most connected genes (top 5%) were identified as hub genes.

### CRISPR/Cas9 plasmid construction and plant transformation

The sgRNA target sequences for the *BpWOX* genes were designed in the online tool CRISPR-P (http://crispr.hzau.edu.cn/CRISPR/), and then the sgRNA target sequences were built into the *pRGEBB1* vector, which was a *pRGEB32* vector modified by changing the hygromycin resistance to herbicide resistance. Then, the vector was introduced into *Agrobacterium tumefaciens* LBA4404 for paper mulberry transformation.

For paper mulberry transformation, leaves from aseptic seedlings were cut into 0.5 cm × 0.5 cm pieces, and the leaf pieces were put into MS liquid medium (including resuspended *Agrobacterium* LBA4404) for ~10 min, and filter papers were used to dry the leaf pieces. Then, the leaf pieces were placed on the induction medium (C1) for 2 days in the dark. Subsequently, the leaf pieces were transferred to the differentiation medium (C2) containing 5 mg/L glufosinate-ammonium, and were cultured under light for 2–3 weeks to induce resistant calluses. Then, the resistant calluses were transferred to the screening medium (C3) with 10 mg/L glufosinate-ammonium to induce buds. Until the putative transgenic buds grew to around 2 cm, they were placed in subculture medium with 10 mg/L glufosinate-ammonium.

To identify the positive transgenic lines, the genomic DNA was extracted by the CTAB method for PCR of the *Bar* gene. Then, the DNAs of putative transgenic lines were amplified and sequenced using specific primers containing target sites (Supplementary Data Table S16), and the mutant lines were identified after comparing them with the sequencing results and the target gene sequences. Finally, the mutant and WT lines were planted and photographed in the greenhouse after root culture. RNA-seq was performed on the WT and *CR-wox* mutant lines, and differential expression gene analysis and KEGG pathway analysis were carried out following the methods presented above.

### Real-time quantitative PCR

To explore the expression patterns of candidate genes among the EL, PL, and NL, the total RNA was extracted from fresh young leaves using the Plant RNA Extraction Kit (TaKaRa, Beijing, China) and reverse-transcribed to cDNA with a PrimeScript RT Reagent Kit (Takara, Beijing, China). Real-time quantitative PCR experiments were conducted using the SYBR PrimeScript RT-PCR Kit (Takara, Beijing, China) following the manufacturer’s protocol, and the gene *BpGAPDH* served as an internal control. The relative expression levels of genes were calculated with the 2^−△△Ct^ method [74]. The sequences of primers used in this study are given in Supplementary Data Table S16.

## Supplementary Material

Supplementary_Figures_uhad212

Supplementary_Tables_uhad212

## Data Availability

The raw re-sequencing data can be found in the NCBI database under the project accession numbers PRJNA974956 and PRJNA870972. The other supplementary figures and tables are summarized in the Supplementary Data files.
