# Supplementary material for: Integrating genome-wide association and transcriptome analysis to provide molecular insights into heterophylly and eco-adaptability in woody plants
Source: Hortic Res. 2023 Nov 17;10(11):uhad212. doi: 10.1093/hr/uhad212 (PMC10689056; doi:10.1093/hr/uhad212)
Supplement: Supplementary_Figures_uhad212 [file supplementary_figures_uhad212.pdf]

Supplementary Figures

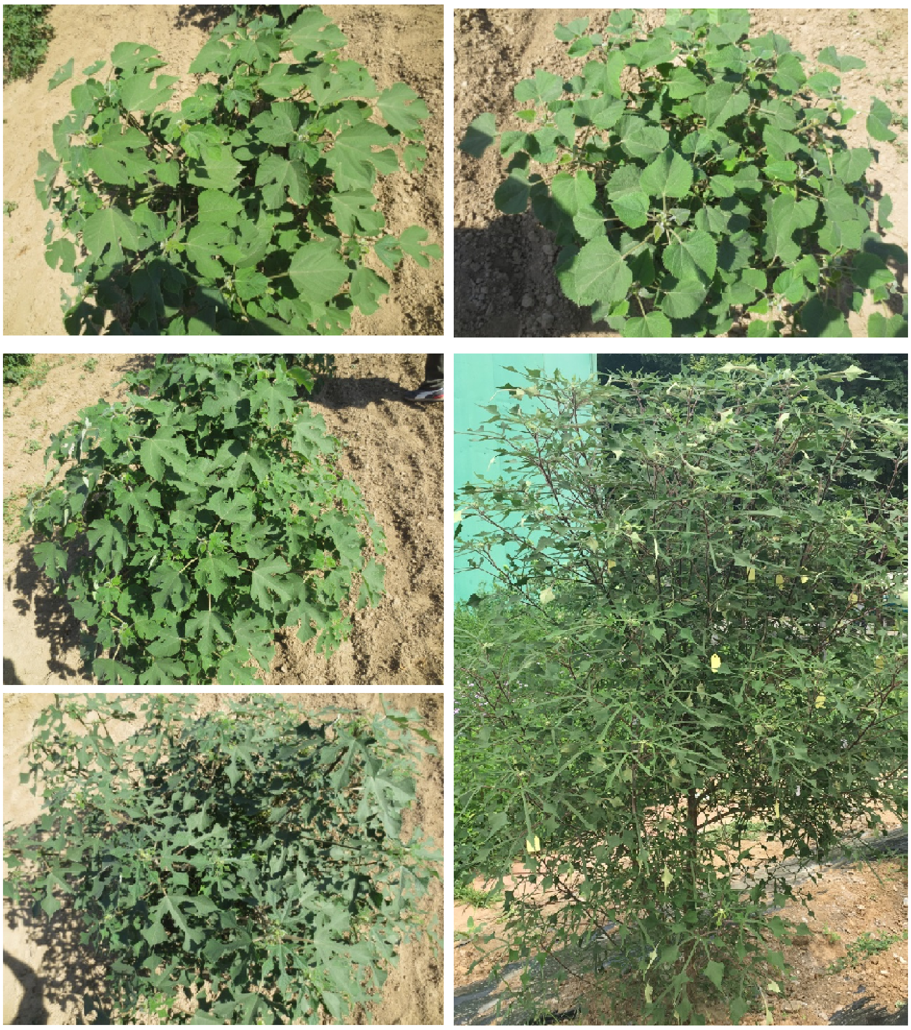

Figure S1 The leaf phenotypic variation and heterophylly in paper mulberry.

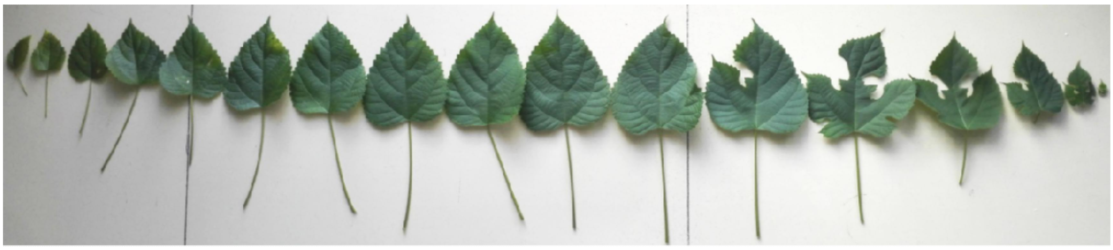

Figure S2 The morphology of the leaves from the same plant of paper mulberry.

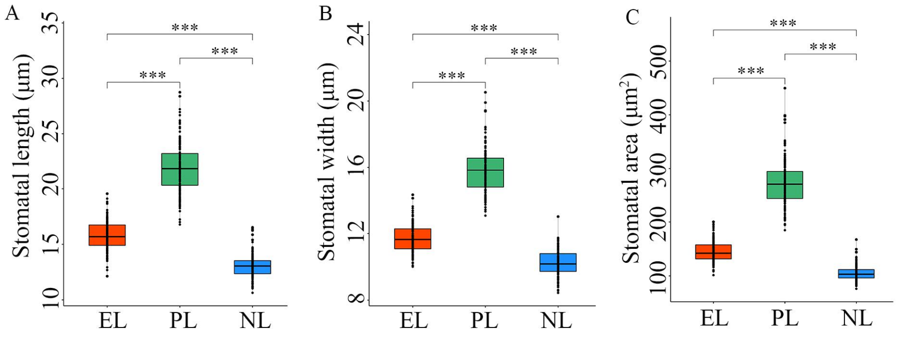

Figure S3 The investigation result of the stomatal indexes. Asterisks indicate the significant differences as analysed by Student's *t*-test (*** represents $P < 0.001$). (A) Stomatal length. EL: entire leaf; PL: palmatisect leaf; NL: natural leaf. (B) Stomatal width. (C) Stomatal area.

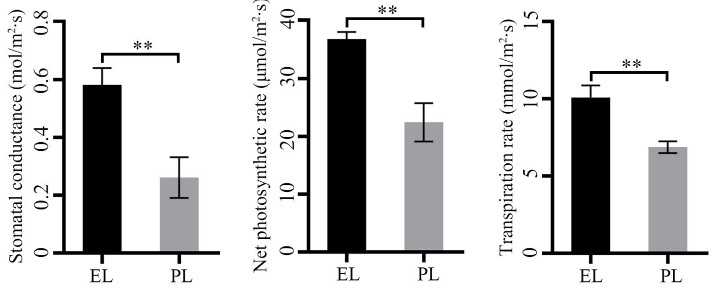

Figure S4 The investigation result of the photosynthetic parameters. Asterisks indicate the significant differences as analysed by Student's *t*-test (** represents $P < 0.01$). EL: entire leaf; PL: palmatisect leaf.

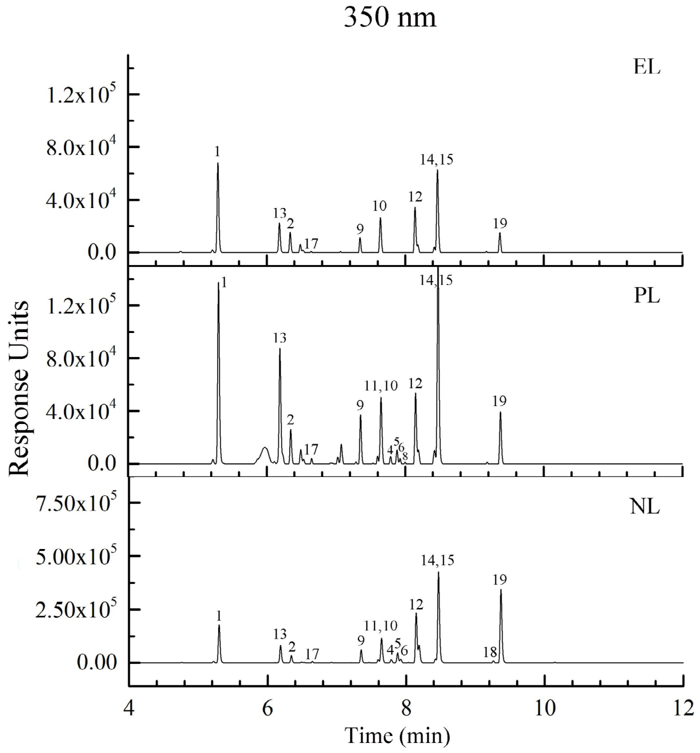

Figure S5 The UPLC chromatograms of heterophyllous leaves in 350 nm. EL: entire leaf. PL: palmatisect leaf. NL: natural leaf.

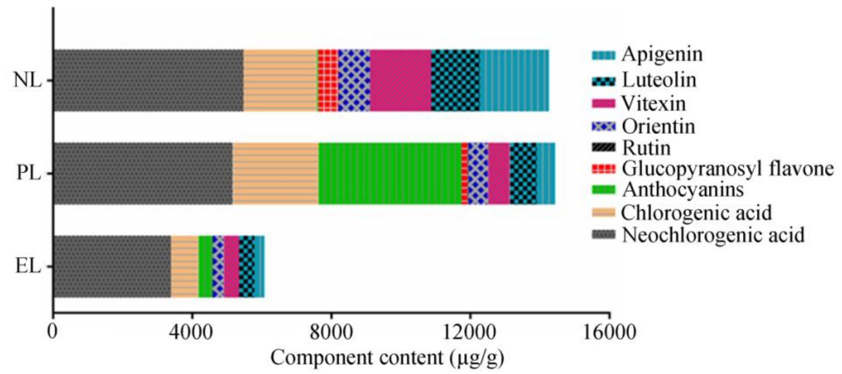

Figure S6 Quantitative analyses of phenolic compound in three type leaves. EL: entire leaf. PL: palmatisect leaf. NL: natural leaf.

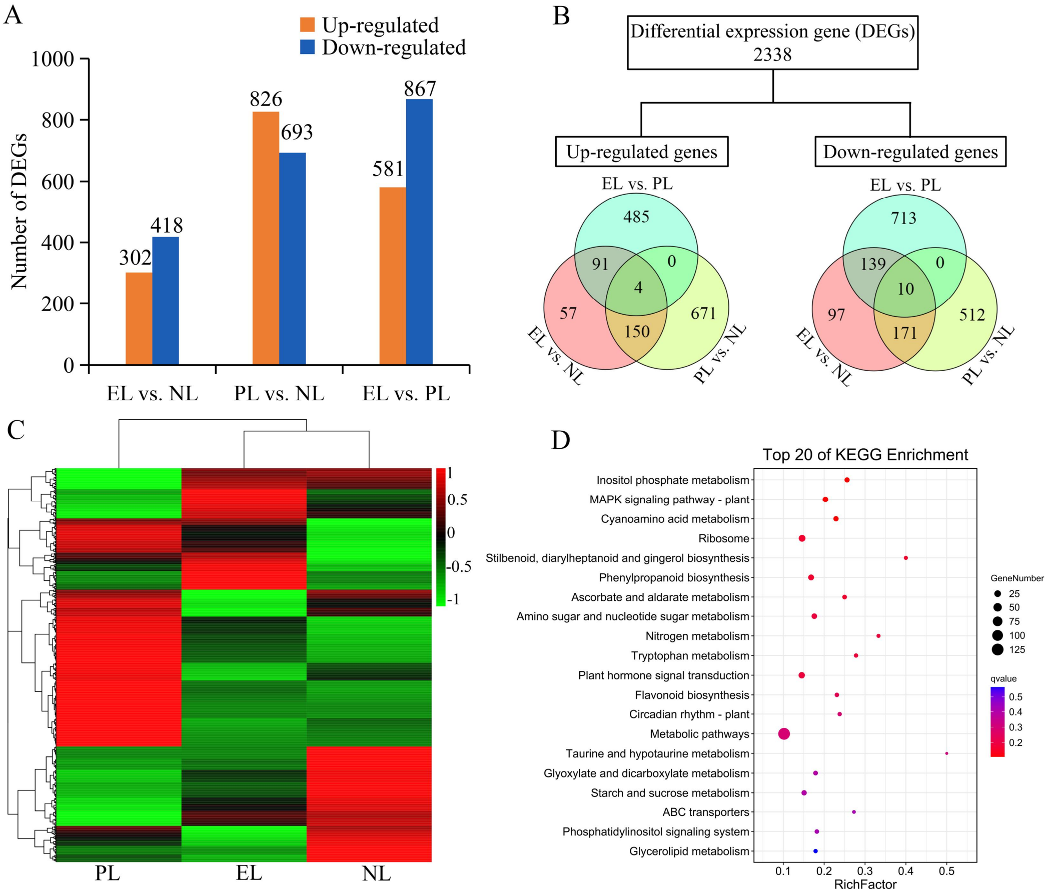

Figure S7 Analysis of differentially expressed genes (DEGs) in three paper mulberry materials with different leaf types. (**A**) The number of DEGs. PL: palmatisect leaf, EL: entire leaf, NL: natural leaf. (**B**) Venn diagram analysis of DEGs. (**C**) Heatmap showed the relative expression levels of total DEGs. (**D**) The KEGG enrichment analysis among DEGs.

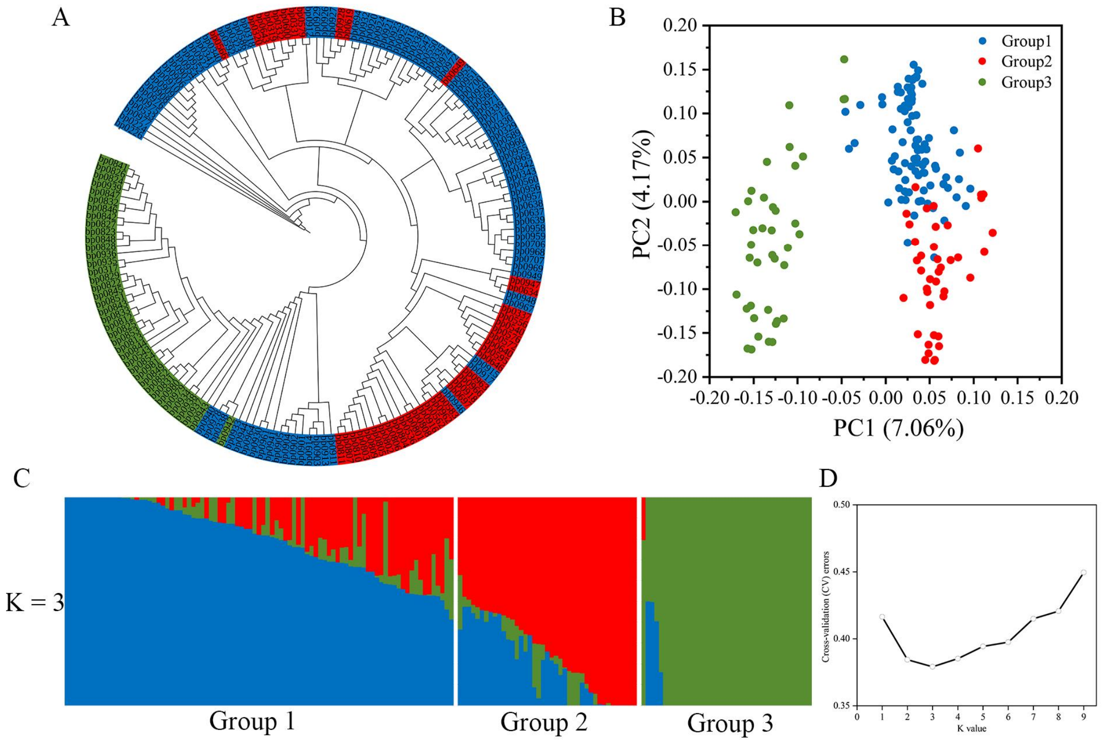

Figure S8 Phylogenetic relationship and population structure of 170 paper mulberry individuals. (A) Phylogenetic tree of all individuals constructed from the whole-genome SNPs. (B) Principal component analysis of 170 paper mulberry individuals using genome-wide SNPs. PC1: first principal component; PC2: second principal component. (C) Population structure analysis estimated by ADMIXTURE (version 1.3.0). (D) The cross-validation error estimate plot used for selecting the optimal K value of the population structure. K = 3 explains the best model for these 170 individuals (when K = 3, the CV error is smallest).

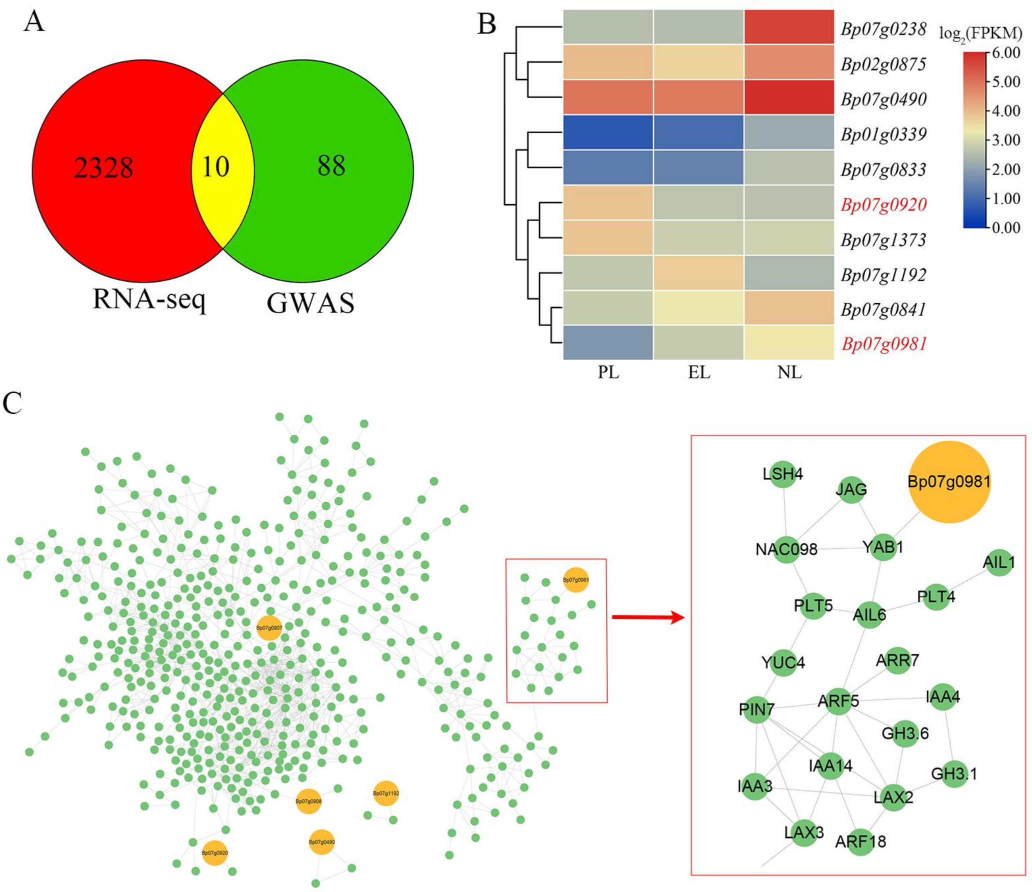

Figure S9 Integrated analysis of the genes identified by GWAS analysis and comparative transcriptome analysis. (A) The venn diagram of the differentially expressed genes (DEGs) identified by RNA-seq and candidate genes identified by GWAS analysis. (B) The expression pattern of genes identified by both the GWAS analysis and comparative transcriptome analysis. (C) Protein-protein interaction analysis of genes identified by GWAS analysis and comparative transcriptome analysis. The yellow colors represent the proteins encoded by crucial genes (*Bp07g0920*, *Bp07g0981*, *Bp07g0490* and *Bp07g1192* were common genes identified by both the GWAS and comparative transcriptome analysis, while *Bp07g0907* and *Bp07g0908* were the key genes with important genetic variation), and the green colors represent the proteins encoded by the other genes identified by GWAS analysis and comparative transcriptome analysis.

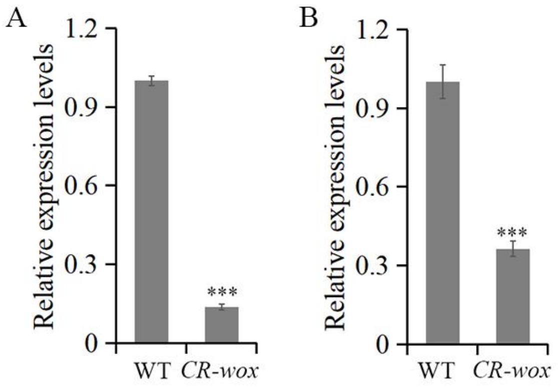

Figure S10 Relative expression levels of *BpWOX1* (*Bp12g1344*) and *BpWOX3* (*Bp09g1360*) in the paper mulberry wild-type (WT) and CRISPR/Cas9-edited *wox* mutant (*CR-wox*) lines. (**A**) *BpWOX1* (*Bp12g1344*). (**B**) *BpWOX3* (*Bp09g1360*). Asterisks indicate the significant differences as analysed by Student's *t*-test (*** represents $P < 0.001$).

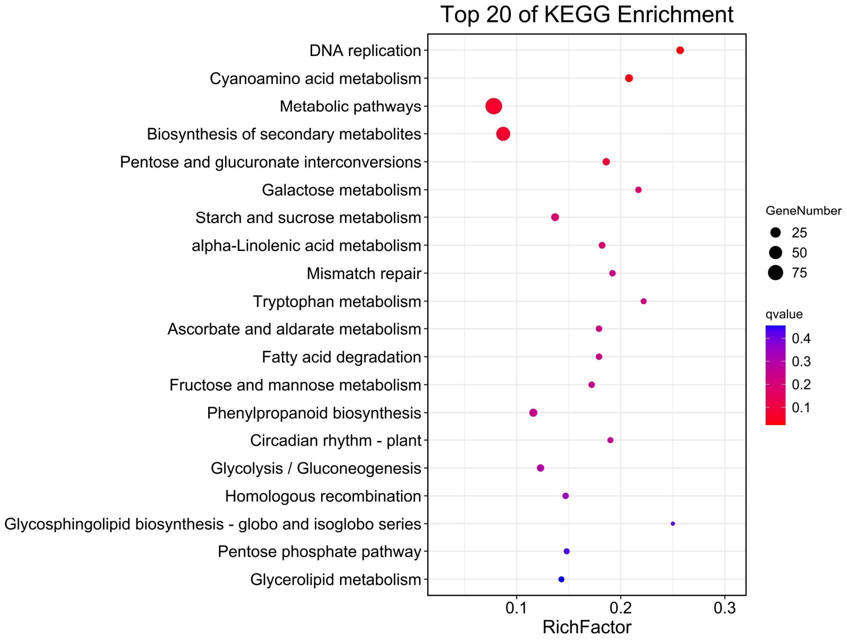

Figure S11 The KEGG enrichment analysis among differentially expressed genes between the the paper mulberry wild-type (WT) and CRISPR/Cas9-edited *wox* mutant (*CR-wox*) lines.
